# Diversity Ideologies and the Subtle Dehumanization of Indigenous Peoples in the Philippines

**DOI:** 10.3390/bs16071142

**Published:** 2026-07-07

**Authors:** Allan B. I. Bernardo

**Affiliations:** Department of Psychology, De La Salle University, Manila 1004, Philippines; allan.bernardo@dlsu.edu.ph

**Keywords:** indigenous peoples, dehumanization, cultural ideologies, diversity ideologies, polyculturalism, multiculturalism, social dominance orientation, outgroup knowledge, Philippines

## Abstract

Indigenous peoples (IPs) in the Philippines continue to be targets of stereotypes and prejudice by non-IP Filipinos. The IPs in the Philippines are often described as being dehumanized by their social conditions and by the social practices of the dominant social group. This study explores the subtle dehumanization of three IPs (Igorots, Mangyans, Lumads) by measuring the attribution of traits associated with human nature and whether subtle dehumanization is negatively associated with two diversity ideologies: multiculturalism and polyculturalism. A sample of 534 non-IP Filipinos were asked to rate whether positive traits that were previously ascribed as high or low characteristics of human nature (HN) were typical of Filipinos in general, of Igorots, Mangyans, and Lumads. Participants’ outgroup knowledge of each IP group, social dominance orientation, egalitarianism, essentializing race, polyculturalism, and multiculturalism were measured. The results show that high-HN traits were attributed less to IP groups compared to Filipinos, but low-HN traits were attributed more to Igorots. Three relative dehumanization indices (RDIs) were computed; across all IP groups, RDIs were associated with lower polyculturalism but not multiculturalism. The implications of how polyculturalism relates to less subtle dehumanization of IP groups in the Philippines are discussed.

## 1. Introduction

Indigenous peoples (or IPs) of the Philippines are estimated to comprise at least 10% of the Philippine population (Bengwayan-Anongos, 2022), but because most non-IP Filipinos lack contact and understanding of the lives of Filipino IPs, IPs continue to be the target of negative stereotypes and prejudice (Blanco, 2024; Salvador-Amores, 2020). The IPs encompass numerous small communities that still retain much of their cultural norms and practices from before the Philippines was colonized. Many of the small IP communities are identified as belonging to IP groups like the Igorots, whose ancestral domains are in the Cordillera Mountain Range in Northern Luzon. Another IP group is the Mangyans, who live in the interior mountainous areas of Mindoro Island. In the Southern Philippine Island of Mindanao, the Lumads comprise groups of IPs who reside in remote areas like forests, coastal areas, and hinterlands. These IPs reside in geographically very isolated areas, which makes access to basic social services beyond their reach, and many IP communities are among the poorest in the Philippines (Bengwayan-Anongos, 2022; United Nations Development Programme, 2010). Many scholars have written about the dehumanizing conditions in the lives of various Philippine IPs (Eduardo & Gabriel, 2021; Violon, 2024), and how some social practices tend to dehumanize Philippine IPs (Blanco, 2024), for example, when the basic education curriculum marginalizes, or worse, erases the IPs’ cultural norms and experiences in the social studies and civics curriculum (Eduardo & Gabriel, 2021). Some school textbooks (Blanco, 2024) and social media posts (Domogen, 2024) also carry negative stereotypes of IPs as less civilized or even uncivilized, contributing to non-IPs’ prejudices against IPs.

This study is an attempt to find quantitative evidence for the subtle dehumanization of Philippine IPs using a paradigm developed by social psychologists (Bain et al., 2009; Haslam & Stratemeyer, 2016) that assumes that dehumanization may involve unconscious implicit cognitions and behaviors. This subtle form of dehumanization is distinct from blatant forms that deliberately and consciously treat certain groups of people as non-human or as being closer to primates and animals (Kteily et al., 2015). The study further explores whether any forms of subtle dehumanization are associated with two diversity ideologies—multiculturalism and polyculturalism.

The next subsections provide a brief background of IPs in the Philippines, followed by a discussion on subtle dehumanization as a form of implicit prejudice for marginalized outgroups like IPs. The two diversity ideologies of multiculturalism and polyculturalism are then discussed as beliefs that are associated with less prejudice towards outgroups.

### 1.1. Indigenous Peoples in the Philippines

IPs in the Philippines are ethnic groups that inhabited the Philippine islands before the Spanish colonized these islands and maintained partial isolation because they stood their ground against the colonizers or withdrew to the hinterlands as the colonizers encroached on their lands (Molinas, 2004). Gradually, non-IP Filipinos started migrating and settling in IP lands, and this was intensified during the American colonial government, which supported the seizure of Ips’ communal lands through the legislature. These colonial policies reconfigured social and political boundaries that resulted in the displacement of IPs from their traditional ancestral domains (Molinas, 2004). The displacement resulted in the IPs resettlement in geographically isolated areas, which allowed the IPs to retain much of their precolonial cultural norms and practices but also isolated them from mainstream social and economic activities (United Nations Development Programme, 2010). When the Philippines gained independence, the early Philippine national government’s many policies favored business and commercial interests that coveted the habitats of the IPs because these contained valuable natural resources; these policies resulted in continued displacement and loss of their ancestral lands (Molinas, 2004; United Nations Development Programme, 2010).

Today, the National Commission of Indigenous Peoples (NCIP) identifies 95 distinct tribes of IPs, many of which are grouped into larger groupings. Three of the largest groupings are the Igorots, the Mangyans, and the Lumads. The Igorots are the IPs in six provinces in Northern Luzon, specifically in the Cordillera Mountain Range. Some of the ethnolinguistic tribes under the grouping are the Bontoc, Ibaloi, Ifugao, Isneg, Kalinga, Kankanaey, Tingguian, and Yapayao (Labrador, 1997). The word Igorot means “mountain people,” and the Igorots are pejoratively referred to by non-IPs as mountain dwellers stereotyped as simple, uneducated, or backward. The Mangyans are not as numerous as the Igorots, but they also come from the mountains and foothills of the island of Mindoro, south of Luzon. Some of the ethnolinguistic tribes in this grouping are the Alangan, Batangan, Buhid, Hanunuo, Iraya, Rataganon, Tadyawan, and Tawbuid (Barbian, 1977). The Mangyans are often referred to in popular culture as being reclusive; the word Hanunuo means “true” or “pure,” which expresses the group’s sense of identity. The Lumads represent the largest grouping of IPs in the Philippines, which includes the Bagobo, Banwaan, B’laan, Bukidnon, Dibabawon, Higaanon, Mamanua, Mandaya, Manobo, Mansaka, Manguangan, Subanen, Tagkaolo, Talandig, Tiboli, Tiruray, and others. These IPs are from the hilly regions of almost all provinces of Mindanao. The word Lumad means “born and grown in the place” in the Visayan language (Molinas, 2004).

Most non-IP Filipinos have minimal or no direct contact with different IPs in the Philippines. As such, non-IPs’ knowledge about the IPs is filtered through depictions in media (Domogen, 2024; Fong, 2007; Salvador-Amores, 2009; Tindaan, 2010) and in educational materials (De Los Reyes, 2021), which often misrepresent the diverse lives and experiences of the IPs. Research on such materials indicates a tendency to present simplifications and cliches associated with primitiveness and backwardness that tend to strengthen negative stereotypes of IPs. Such stereotypes shape how non-IP Filipinos perceive IPs (Tindaan, 2010), but also the social identity and self-esteem of IPs (Botangen et al., 2017). Many IPs share experiences of being targets of stereotypes and prejudice among fellow Filipinos (Paredes, 2019; Salvador-Amores, 2020).

### 1.2. Dehumanization of Outgroups

Stereotyping and prejudice of outgroups and cultural minorities like the IPs in the Philippines come in different forms. One of the most noxious forms involves denying the humanity of outgroups or overlooking that outgroups belong to the same superordinate category of humans, together with one’s ingroup. This form of prejudice is called dehumanization in the social psychology literature (Haslam & Loughnan, 2014); the blatant forms of dehumanization involve likening members of the outgroup to animals (Kteily et al., 2015), which seems to be pervasive in contemporary social experiences (Kteily & Landry, 2022). On the other hand, there might even be more pervasive yet subtle forms of dehumanization, such as the tendency to downplay the capacity of an outgroup to feel, think and plan (Gray et al., 2007) or demeaning outgroup members’ drive to meet more complex psychological needs (Schroeder & Epley, 2020); in both cases, dehumanization involves denying aspects of humanness to the outgroup. Another example of a subtle form is the tendency to associate images of IPs with animal-related stimuli, more readily so than with people from modern societies (Saminaden et al., 2010).

Another form of subtle dehumanization relates to the tendency to downplay categories of traits that are associated with full humanity (Haslam & Loughnan, 2014; Haslam & Stratemeyer, 2016). A distinction is made between traits that reflect human nature (HN), like curiosity and interpersonal warmth, and traits that reflect human uniqueness (HU), such as rationality and civility (Haslam et al., 2005). High-HN traits are attributed to humans but are not assumed to be unique to humans (as they are shared by some non-human species), whereas high-HU traits are seen as attributes only of humans but are not necessarily part of human nature. Studies have identified traits that are both High HU and High HN (e.g., ambitious, analytic, imaginative) and traits that are high HU but low HN (broad-minded, humble, polite) (Haslam & Bain, 2007). One subtle form of dehumanization is the tendency to attribute high-HN traits more to the self than to others, which is referred to as self-humanizing (Haslam et al., 2005), which implicitly means attributing less humanness to others. Some researchers have suggested that there seems to be no evidence of a similar effect involving HU traits (Cortes et al., 2005; Haslam et al., 2005) and that subtle dehumanization involves differences in attribution only of HN traits. However, in their cross-cultural study, Bain et al. (2009) found that Anglo-Australians attributed fewer HN traits, but more HU traits, to ethnic Chinese, while ethnic Chinese attributed fewer HU traits to Anglo-Australians. Thus, it is possible that there are different forms of subtle dehumanization across groups that involve either or both HN and HU traits.

### 1.3. Diversity Ideologies

Previous research on subtle dehumanization related to the attribution of HN and HU traits suggests that it may be a reflection of a range of psychological processes, such as egocentric cognitions, lack of empathy, and the more abstract construal of others (Kteily & Landry, 2022). Ideological and system-justifying beliefs have also been established as predictors of dehumanization in different intergroup contexts (Kteily et al., 2015; Trounson et al., 2015). In intercultural research, different diversity ideologies have been associated with different cognitions and behaviors towards outgroups. Much work has been done involving three ideologies: colorblindness, multiculturalism, and polyculturalism, each of which has distinct positive and negative associations with outgroup attitudes (see Rosenthal & Levy, 2010, for review). The colorblind ideology minimizes the importance of cultural identity and race by emphasizing the treatment of people as unique individuals based on their own qualities and behaviors (Wolsko et al., 2000), but its associations with intercultural attitudes are mixed. Among members of the dominant cultural group, colorblindness reduced explicit racial/ethnic bias in the short term, but increases later implicit prejudice (Correll et al., 2008 Richeson & Nussbaum, 2004).

In the past two decades, there has been more interest in two diversity ideologies that acknowledge the importance of cultural identities, instead of being blind to these: multiculturalism and polyculturalism. Multicultural ideology argues that cultural differences should be acknowledged, respected, and celebrated (Plaut, 2010). Priming multicultural ideologies tends to be associated with less outgroup prejudice (Richeson & Nussbaum, 2004; Sparkman et al., 2016; Stevens et al., 2008), but was also associated with greater stereotyping (Ryan et al., 2010) and exclusion (Plaut et al., 2011). Polycultural ideology, like multiculturalism, recognizes culture-based group differences but emphasizes the mutual interactions, connections, and therefore, similarities between cultural groups (Rosenthal & Levy, 2012). An important presumption of polyculturalism is that culture is not viewed as a fixed or rigid concept, and that cultures are dynamic and constantly changing because of intercultural interactions over time (Morris et al., 2015). Like multiculturalism, polyculturalism has been associated with more positive intercultural attitudes (Bernardo et al., 2013; Rosenthal et al., 2016, 2019), including towards outgroup cultural minorities (Batkhina et al., 2022; Menadue et al., 2021; Rosenthal et al., 2015). Pertinent to the current study, a previous study found the endorsement of polyculturalism to be positively associated with more positive attitudes towards cultural minorities and indigenous people in the Philippines (Bernardo, 2024).

There are important differences between how multiculturalism and polyculturalism relate to intercultural or intergroup processes. For example, while multiculturalism tends to be associated with essentializing race, polyculturalism is not (Bernardo et al., 2016). Priming multiculturalism tends to heighten negative views about cultural accommodation, while polyculturalism does not (Cho et al., 2017). Polyculturalism is also associated with ethnocultural empathy, higher quality intercultural contact, and lower cultural identity clarity (Virgona & Kashima, 2021), and creative problem solving (Cho et al., 2018), but multiculturalism is not. Thus, although both multiculturalism and polyculturalism are diversity ideologies associated with positive intercultural outcomes, their relationships with specific intercultural processes are not always the same.

### 1.4. The Current Study

The aims of this study are twofold. The first is to determine whether non-IP Filipinos dehumanize IPs in the Philippines using a paradigm that measures subtle dehumanization that involves the attribution of HN traits (Bain et al., 2009). The second is to determine if such subtle dehumanization is negatively associated with either of two diversity ideologies: multiculturalism and polyculturalism.

Three major IP groups are the focus of the current study: Igorots, Lumads, and Mangyans. Subtle dehumanization is defined as the tendency to attribute less high-HN traits and to attribute more low-HN traits to the IP groups relative to the group of Filipinos. Participants are asked to indicate whether they think different traits are descriptive of each group; four traits are high-HU/high-HN traits, and four are high-HU/low-HN traits based on previous studies (Haslam et al., 2005; Bain et al., 2009). Only high-HU traits were included because some research suggests that this form of subtle dehumanization is more likely to involve the denial of HN to outgroups (Haslam et al., 2005), so HU traits were not varied. All eight traits were positive to avoid causing offense and discomfort when using negative traits. The details of the dehumanization measure are explained in Section 2. The first hypothesis had two versions:

- H1a: High-HU/high-HN traits would be attributed less to the three IP groups compared to the Filipino group.
- H1b: High-HU/low-HN traits would be attributed more to the three IP groups compared to the Filipino group.

Both multiculturalism and polyculturalism were hypothesized to be negatively associated with the subtle dehumanization of IPs in the Philippines. The two diversity ideologies that emphasize the importance of cultural identity and cultural differences were hypothesized to be associated with a weaker tendency to dehumanize IPs. One study found polyculturalism to be positively associated with attitudes towards Filipino IPs, but that study did not measure multiculturalism (Bernardo, 2024); thus, it was important to examine the two diversity ideologies in one study. It was also important to control for the possible influence of other factors known to be associated with outgroup attitudes, foremost of which was outgroup knowledge, which is a strong predictor of less negative attitudes towards outgroups (Long et al., 2023; Zagefka et al., 2017). The other control variables included were those controlled in similar studies (Bernardo, 2024; Bernardo et al., 2019) involving outgroups or minority groups in the Philippines: social dominance orientation, egalitarian beliefs, and essentializing race. The second hypothesis was:

- H2: Endorsement of multiculturalism and polyculturalism will be negatively associated with the subtle dehumanization of Filipino IPs, after controlling for factors associated with intercultural outcomes.

## 2. Materials and Methods

### 2.1. Participants and Procedures

The participants were 534 university students from Metro Manila who provided their informed consent. All participants were at least 18 years old (M = 19.37, SD = 2.03); 201 self-reported as male, 332 as female, and 1 preferred not to respond. All grew up in the Philippines, except for 3 who had Filipino parents but grew up and completed high school in a foreign country. None of the participants belonged to any IP groups in the Philippines.

After providing their informed consent, participants were presented with a questionnaire that contained background information questions, followed by the relevant scales in one fixed order. The questionnaire included a self-esteem scale and items that referred to other minority groups in the Philippines that were not included in the current analysis.

### 2.2. Materials

All the materials were presented in English, the medium of instruction in Philippine higher education institutions. Except when indicated, participants responded to the items in the measures using a scale from 1 (strongly disagree) to 6 (strongly agree).

Dehumanization measure: The participants were asked to share their perceptions of the four groups: Filipino people, Igorots, Lumads, and Mangyans, using different adjectives. The instructions were: “You will now be asked to think about Filipino people as a whole and some cultural minority groups in the Philippines. Please rate whether each of the given adjectives is typical or not of the group.” The participants were asked to respond to each adjective using the scale from 1 (not at all typical) to 6 (highly typical). The adjectives were selected from those identified in Bain et al.’s (2009) study as being High Human Uniqueness and High Human Nature (High HU/High HN) traits: ambitious, analytic, imaginative, passionate, and High Human Uniqueness and Low Human Nature (High HU/Low HN) traits: broad-minded, humble, polite, conscientious. Only positive traits were included in the question. In the results section, the computation of a relative dehumanization index is described.

Diversity ideology scales: The five-item polyculturalism scale and five-item multiculturalism scale developed by Rosenthal and Levy (2012) were used. Sample items include: “Different racial, ethnic, and cultural groups influence each other” (polyculturalism) and “All cultures have their own distinct traditions and perspectives” (multiculturalism). For the current sample, both scales had good reliability; polyculturalism: McDonald’s ω = 0.89; Cronbach’s α = 0.89; multiculturalism: McDonald’s ω = 0.82; Cronbach’s α = 0.83.

Social dominance orientation: Seven items of the short version of the social dominance orientation scale (Pratto et al., 1994) were used; sample item: “Some groups of people are simply not the equal of others”. The scale had adequate reliability: McDonald’s ω = 0.76, Cronbach’s α = 0.75.

Egalitarianism scale: The six-item egalitarianism scale (Katz & Hass, 1988) was used; sample item: “People should help others who are less fortunate than they are.” The scale had adequate reliability: McDonald’s ω = 0.82, Cronbach’s α = 0.82.

Essentializing race scale: The four-item Genetic Lay Theory of Race Scale (No et al., 2008) was used; sample: “To a large extent, a person’s race biologically determines his or her abilities and traits”. The scale had adequate reliability: McDonald’s ω = 0.74, Cronbach’s α = 0.73.

Outgroup knowledge: Participants were given the following instructions: “Please indicate how much knowledge you have about each group. Your knowledge may come from personal experience or from books, newspapers, mass media or social media.” The participants indicated their answer using a scale from 1 (I know nothing about this group) to 6 (I know a lot about this group): The three groups referred to Igorot people, Lumad people, and Mangyan people.

## 3. Results

One participant did not answer the items in the dehumanization measure for Igorots. The missing scores for this participant were imputed by substituting the mean.

### 3.1. Human Nature Traits

The average trait ratings for High HU/High HN and High HU/Low HN for each of the four groups were computed and are summarized in Table 1. Both averages represent ratings on traits that are assumed to be uniquely human, but they differ in the degree to which they are assumed to be natural attributes of humans. Thus, differences in High-HU/High-HN scores may be interpreted as indicating how one group is seen as possessing more or less of the attributes considered to be natural human qualities; whereas differences in High-HU/Low-HN scores may be interpreted as indicating how one group is seen as possessing more or less of the attributes that are not associated with human nature.

The means for each type of trait were analyzed using a repeated measures Analysis of Variance. For the High-HU/High-HN scores, there was a significant main effect of target group, *F* (3, 1596) = 73.52, *p* < 0.001, η^2^ = 0.12. The means were compared using post hoc pairwise comparison of means adjusted for comparing a family of six estimates corrected using the Bonferroni method. As shown in Table 1, High-HU/High-HN traits were more likely to be attributed to the Filipino people as a group, and less so to Igorots and Lumads, and least to Mangyans. The results could be read as indicating that the participants in the study were attributing less of the traits associated with human nature to the three IPs compared to the non-IP Filipinos.

There was also a significant main effect of target group when comparing the High-HU/Low-HN scores, *F* (3, 1596) = 10.73, *p* < 0.0001, η^2^ = 0.02, but the effect size was extremely small. The post hoc pairwise comparison of means indicated that only the scores for Igorots were statistically different from the other means (see Table 1). The pattern suggests that the participants were not likely to differ in their attribution of traits not associated with human nature, except for the Igorots, where the traits were attributed more highly.

So far, the comparison of trait attributions indicates that non-IP Filipinos seem to be denying high human nature traits to the three Filipino IP groups, by attributing them to these groups at lower levels compared to non-IP Filipinos. The data do not indicate that the non-human nature traits are being attributed more to Filipino IP groups, except for the Igorots. So the Igorots are being attributed less high human nature traits but also more low human nature traits. It should be noted, however, that the low human nature traits are also high human uniqueness traits, so they do embody a different aspect of the target groups’ humanness; a comparison of the High-HU/Low-HN scores across groups may not indicate relative dehumanization or lack thereof in a straightforward manner. Thus, there is evidence that there is subtle dehumanization of IPs by the non-IP participants, but more specifically associated with the attribution of less positive High-HU/Low-HN traits.

### 3.2. Relative Dehumanization Indexes (RDIs)

To explore the relationship between subtle dehumanization and the participants’ cultural diversity ideologies, three indexes were created to a relative measure of dehumanization adopting an approach to measuring subtle dehumanization used by Bain et al. (2009). All indices involved comparing the attribution of human traits to the IP group to that of the dominant Filipino group. In all indexes, subtle dehumanization is measured as the degree to which the human traits are ascribed less to the IP than to the Filipino group, using a simple difference score.

The first index, based on the attribution of High-HU/High-HN traits, is a relative dehumanization index, specific to High-HN traits (henceforth, RDI-HHN). This index is the difference score between High-HU/High-HN traits for Filipinos and for each IP group. Higher or more positive RDI-HHNs are assumed to indicate stronger subtle dehumanization by attributing less of high-HN traits.

The second index is based on the attribution of High-HU/Low-HN traits (henceforth, RDI-LHN). This index is computed inversely, as for the RDI-HHN, the difference score between High-HU/Low-HN traits for each IP group and for Filipinos. Higher or more positive RDI-LHNs are assumed to indicate stronger subtle dehumanization by attributing more of the low-HN traits to the IP groups. Earlier, it was noted that the implication of High-HU/Low-HN traits for the dehumanization of IPs is not as straightforward because they also embody HU, which is a different aspect of humanness. This point will be considered when the RDI-LHN results are interpreted.

Also in consideration of the preceding point, the third index is based on the attribution of all High-HU traits (i.e., combining High-HU/High-HN and High HU/Low-HN traits). This index (henceforth, RDI-HHU) is the difference score between High-HU traits for Filipinos and for each IP group. Higher or more positive RDI-HHUs are assumed to indicate stronger subtle dehumanization by attributing less of high-HU traits to IPs.

The descriptive statistics for all the RDIs for each IP group are summarized in Table 2, which shows that most of the modal RDIs for the three groups were zero. This means that in most cases (except for the human uniqueness traits), most participants were more likely not to attribute more or less high human traits to the IP groups compared to Filipinos, and the means are all close to zero.

Three repeated measures ANOVA, one for each RDI, were conducted. For RDI-HHN, a significant main effect of target group was *F* (2, 1064) = 5.55, *p* = 0.004, η^2^ = 0.01, but the effect size was near zero. The post hoc pairwise comparison of means adjusted for comparing a family of three estimates corrected using the Bonferroni method indicated that there was a higher RDI for the Mangyans compared to the Igorots [*M*_diff_ = 0.07, *SE*_diff_ = 0.02, *t*(532) = 3.43, *p* = 0.002] and Lumads [*M*_diff_ = 0.06, *SE*_diff_ = 0.02, *t*(532) = 2.75, *p* = 0.018]. For RDI-LHN, a significant main effect of target group was *F* (2, 1064) = 15.53, *p* < 0.001, η^2^ = 0.03; again, the effect size was near zero. The post hoc pairwise comparison of means adjusted using the Bonferroni method indicated that there was a higher RDI for the Igorots compared to the Lumads [*M*_diff_ = 0.14, *SE*_diff_ = 0.03, *t*(532) = 4.55, *p* < 0.001] and Mangyans [*M*_diff_ = 0.11, *SE*_diff_ = 0.02, *t*(532) = 4.98, *p* < 0.001]. Finally, for RDI-HHU, a significant main effect of target group was *F* (2, 1064) = 10.12, *p* < 0.001, η^2^ = 0.02. The post hoc pairwise comparison of means using the Bonferroni method indicated that there was a higher RDI for the Igorots compared to the Lumads [*M*_diff_ = 0.07, *SE*_diff_ = 0.02, *t*(532) = 3.05, *p* = 0.007] and Lumads [*M*_diff_ = 0.09, *SE*_diff_ = 0.02, *t*(532) = 5.00, *p* < 0.001]. But the small effect size of these differences should be restated.

Table 2 shows that there were a few participants who had high RDIs, suggesting high levels of subtle dehumanization; there were also a few who had negative RDIs, suggesting the attribution of more human nature traits to the IPs. To test the hypothesis regarding the relationship between diversity ideologies and each index of subtle dehumanization of IPs, nine hierarchical regression analyses were conducted, one for each of the three RDIs of the three IP groups. In each analysis, the first model considered age, sex, and three factors known to be associated with outgroup attitudes towards minority groups: outgroup knowledge, social dominance orientation, egalitarianism, and essentializing race. Polyculturalism and multiculturalism were entered in the second model, and the change in the *R*^2^ model was assessed. Data from three participants were not included in the regression analyses because there were missing values for at least one of the outgroup knowledge measures; the missing values were not imputed. (The correlations among all the variables in the regression analysis are summarized in Supplementary Table S1).

The assumptions of normality and homoscedasticity of residuals were checked for each of the regression analyses. Q-Q plots of standardized residuals were generated and visually assessed to show close alignment with the diagonal line, suggesting normality of residuals. The standardized residuals were also plotted against the standardized predicted values, and in all cases, a random cloud of points was found, suggesting homoscedasticity of residuals. Casewise diagnostics were also conducted using Cook’s and Mahalanobis distance, and in all regression analyses, no influential cases were identified. The results of the nine hierarchical regression analyses are summarized in Table 3, Table 4 and Table 5. For efficiency, only the results of the final model are presented in each table (the full model can be viewed in Supplementary Tables S2–S4.

All the regression analyses indicated small but significant *R*^2^s for all three groups, which suggests that there are other factors that might better predict the variation in RDIs or subtle dehumanization in the sample. Nevertheless, the significant regression models allow for an examination of how the two cultural ideologies are associated with the three indices of subtle dehumanization of IP groups in the Philippines.

For the RDI-HHN (see Table 3), among the control variables, outgroup knowledge was only associated with lower subtle dehumanization of the Lumads. Social dominance orientation and egalitarianism were both positively associated with the subtle dehumanization of the Igorots and Lumads. But most pertinent to this analysis, in all three regression models, the inclusion of diversity ideologies resulted in a significant improvement in the model. However, only polyculturalism was consistently negatively associated with lower subtle dehumanization of the three IP groups, with polyculturalism having the largest standardized beta coefficient in all three analyses.

The regression models for the RDI-LHN were mostly nonsignificant (see Table 4). The addition of the cultural ideologies in the hierarchical regression only resulted in an improved model for the Igorots, where polyculturalism negatively predicted the index, but the complete model was still nonsignificant. It was earlier noted that the High-HU/Low-HN traits might not indicate a clear relationship with dehumanization because they also measure attribution of high-HU traits, and so the index is similarly problematic. The nonsignificant results might be capturing the problematic nature of the index.

The results for the dehumanization index that focuses on these high-HU traits (RDI-HHU) follow the same pattern as the ones on high-HN traits (RDI-HHN) (see Table 5). In all three regression models for RDI-HHU, the inclusion of diversity ideologies resulted in a significant improvement in the model, and the *R*^2^s were comparatively higher than with the other two sets of regression models. Again, only polyculturalism was consistently negatively associated with lower subtle dehumanization of the three IP groups, and polyculturalism had the largest standardized beta coefficient in all analyses. Additionally, there was an interesting finding related to the control variables. Essentializing race positively predicted subtle dehumanization in the form of attributing traits associated with high human uniqueness less to IPs, a finding that was not found in the first two sets of analyses. This result suggests that the tendency to attribute uniquely human traits to Filipino IP groups may be associated with the belief that race is biological or genetic in nature. This last result seems to be specific to the subtle dehumanization related to the attribution of traits unique to humans, but not to traits associated with human nature.

## 4. Discussion

### 4.1. Subtle Dehumanization of IP Filipinos

The first objective of the study was to explore whether non-IP Filipinos tend to engage in subtle dehumanization of IP Filipinos, and the results provide preliminary support for this proposition. IP groups were rated as lower in high HN compared to the general group of Filipinos, indicating a tendency to deny IPs the traits associated with human nature. Low-HN traits were attributed more to Igorots; however, given that these low-HN traits are also high-HU traits, the results may not indicate dehumanization. Indeed, the results may actually indicate higher humanization of the Igorots compared to non-IP Filipinos, but one cannot make confident conclusions about this set of traits. The results further show that the subtle dehumanization involving the denial of high HN and high-HU traits to each IP group was negatively associated with polyculturalism, but not with multiculturalism—partially supporting the second hypothesis.

Although advocates of IPs in the Philippines often speak of the dehumanizing conditions of Filipino IPs, there is no study that has measured the psychological aspect of this dehumanization. It is difficult to directly inquire into whether non-IP Filipinos consider IPs as less human than they are, as such expressions of blatant forms of dehumanization are not socially acceptable in Philippine society. The results of the current study document a statistically significant tendency to deny traits commonly associated with HN (e.g., analytic, imaginative, passionate) relative to Filipinos in general, and in the case of the Igorots, to ascribe traits associated with low HN (e.g., broad-minded, polite, conscientious). Note that all the traits that were inquired into were positive traits, so it is not the case that the IPs were being characterized as more negative (a possibility that could not be explored because of a limitation in the measure of the study, see below). Instead, what the results indicate is that, without being aware of it, non-IP Filipinos are associating HN traits to a lesser degree with IP groups than with the social group of Filipinos. This human tendency to dehumanize is not indicative of derogation of IPs because HN does not necessarily mean “good.” But dehumanization is associated with a range of problematic outgroup attitudes and cognitions (see Haslam & Stratemeyer, 2016, for a summary). For example, this subtle form of dehumanization resulted in lower empathy and willingness to help the dehumanized outgroup (Andrighetto et al., 2014). As some have argued, the subtle and blatant forms of dehumanization share similar consequences (Waytz & Schroeder, 2014). And although subtle dehumanization does not relate to the more dire consequences of blatant dehumanization, its consequences tend to relate to acts of omission towards the dehumanized groups: forgetting or ignoring their names, overlooking their needs, and discounting their feelings (Waytz & Schroeder, 2014).

We could surmise that similar behavioral tendencies are directed by non-IP Filipinos to Filipino IPs, but the conjecture will need to be verified in future empirical studies. However, it is important to acknowledge a possible limitation of the current study that relates to the paradigm to measure subtle dehumanization. The attribution of HU and HN traits has been an accepted method to assess how people may be denying the humanity of specific social groups without knowing that they are doing so (Haslam & Stratemeyer, 2016; Kteily & Landry, 2022). The study assumed that Filipinos would also associate the traits with HN and HU. The traits associated with high and low HU and HN were determined from a study involving Australian participants (Haslam et al., 2005), but later studies showed similar ratings across cultural samples (Bain et al., 2009). Nevertheless, the possibility that traits associated with HU and HN might be defined differently among Filipinos. Therefore, future research might need to use sets of traits that are validated with Filipino samples. Such studies can also use other measures of subtle dehumanization to complement the trait-based approaches.

Another important limitation of the dehumanization measure relates to the use of only positive traits to characterize the different IP groups, a decision that was made to avoid the possibility of causing offense and discomfort among the respondents. It was not the intention of the study to suggest that attributing less positive human traits is the only or the most important expression of dehumanization. Indeed, the choice of the specific form and measure of dehumanization in the study does not fully capture the range of cognitive, affective, and behavioral expressions of dehumanization, many of which involve the processing of negative traits, perceptions, attributions, affects, and behaviors.

### 4.2. Diversity Ideologies as Predictors of Subtle Dehumanization of IPs Filipinos

Research and theory suggest a range of motivations for subtle and blatant forms of dehumanization. One view assumes that emotions like disgust and contempt motivate dehumanization (Buckels & Trapnell, 2013; Hodson & Costello, 2007), but another view suggests that dehumanization arises from the anticipation that empathizing with the target group will be emotionally exhausting (Cameron et al., 2016; Diniz et al., 2019). But most theories assume that dehumanization is motivated by ethnocentrism and ingroup protection (Koval et al., 2012; Leyens et al., 2007). In this regard, two diversity ideologies that are assumed to weaken these psychological processes were studied as predictors of subtle dehumanization, and other related intergroup variables were included as covariates. The results only supported the hypotheses that polyculturalism negatively predicted subtle dehumanization, but multiculturalism was not a significant predictor. The non-significant results related to multiculturalism recall the findings that multiculturalism has also been associated with greater stereotyping (Ryan et al., 2010) and exclusion (Plaut et al., 2011) of out-groups, and even essentializing race (Bernardo et al., 2016). The result also resonated with an earlier finding that immigrants who pursue separatism and preserve their cultural boundaries were more likely to dehumanize the citizens of their host nation (Miranda et al., 2014).

The same study found that immigrants who sought to integrate with the host culture were less likely to dehumanize the host citizens, about which the researchers concluded that using inclusive social categories instead of rigid intergroup categories reduces dehumanization (Miranda et al., 2014). This aligns with some core assumptions of the polycultural ideology, as polyculturalism emphasizes that cultures are mutually interconnected and that cultural boundaries are fluid and dynamic (Morris et al., 2015). Polyculturalism is also associated with intergroup processes that are opposed to the ethnocentric and ingroup protective motivations of dehumanization. For example, polyculturalism is associated with lower perceived outgroup threat to ingroup power and status (Osborn et al., 2020), stronger intercultural empathy (Virgona & Kashima, 2021), appreciation for and comfort with diversity (Rosenthal & Levy, 2012), openness to cultural fusion (Cho et al., 2018) and cultural accommodation (Cho et al., 2017). Of the two cultural ideologies, polyculturalism implies the notion that cultural and cultural boundaries change, which aligns more strongly with the notion of more inclusive social categories that reduce dehumanization (Miranda et al., 2014). It may be tempting to suggest that polyculturalism seems to be the diversity ideology that has the potential of moderating the tendency to dehumanize IPs in the Philippines in subtle ways; however, the results of the study are merely correlational in nature, and there is no evidence that the core beliefs of polyculturalism are causing weaker tendencies to dehumanize IP groups in the Philippines. The causal relationship between any diversity ideology and dehumanization can only be established through experimental and/or longitudinal research studies.

It should also be noted that although polyculturalism explained a significant portion of variation in two measures of dehumanization (RDI-HHN and RDI-HHU), the regression models showed small effect sizes associated with diversity ideologies. Across the three regression models, adding the two diversity ideologies in the model resulted in small but significant improvements in the model (Δ*R*^2^ = 0.03 to 0.04 for RDI-HHN; (Δ*R*^2^ = 0.06 for RDI-HHU), and the complete regression models, although significant, accounted for only small portions of the variance in subtle dehumanization (*R*^2^s = 0.07 to 0.11). Thus, it is very likely that there are other, more important factors that play a role in the subtle dehumanization of IPs in the Philippines (see Karantzas & Simpson, 2026), and one should be careful not to overestimate the role of diversity ideologies in subtle dehumanization. The significant positive association between SDO and subtle dehumanization involving HN traits suggests that there are hierarchical intergroup factors that are in play. However, the significant positive associations between egalitarianism and subtle dehumanization also suggest that there might be positive affective dimensions to the phenomenon, as well, consistent with the view that the anticipated emotional exhaustion arising from empathizing with the target group (Cameron et al., 2016; Diniz et al., 2019) is also a factor. For dehumanization that involves HU traits, essentializing race was a significant positive predictor. Recall that HU traits are assumed to be qualities that are only found in humans (e.g., civility, rationality) but are not necessarily part of human nature. Essentializing race is associated with assuming more rigid boundaries between different cultural groups, and it may even indicate some conceptual associations with blatant forms of dehumanization (Landry et al., 2022), which assume that some cultural or racial groups do not possess these uniquely human traits. This possible relationship between essentializing race and the denial of HU traits to outgroups can be further explored in future research.

Future research on the associations between cultural ideologies and dehumanization should also be studied using more heterogeneous samples. The current study only had university students as participants, who may have held more open-minded and liberal ways of thinking compared to most other non-IP Filipinos. The results may not generalize to the non-IP Filipinos, whose cultural ideologies, outgroup knowledge about IPs, and other intergroup beliefs may be more traditional or conservative.

Nevertheless, the negative associations between endorsement of polyculturalism and this subtle form of dehumanization were observed after statistically controlling for the possible role of these other factors. It is very likely that the more flexible thinking about cultural boundaries and cultural groups is opposed to the tendency towards ethnocentric thinking that seems to underlie subtle (and also blatant) dehumanization. If future research can provide evidence for the causal role of polycultural ideology in reducing dehumanization, the core ideas of polyculturalism could be the basis of possible interventions to humanize non-IP Filipinos’ perceptions of IPs. Previous research shows that experiences and information that underscore similarities between groups that are in conflict help humanize the outgroup (McDonald et al., 2017; Saguy et al., 2015), and polyculturalism emphasizes the similarities between groups that arise from the mutual influence and connections. Similarly, migrants who aim to assimilate with instead of to separate from the host culture are also less likely to dehumanize the host culture (Miranda et al., 2014). Such openness, flexibility, and less rigidity as regards cultural boundaries is another core idea in the polycultural ideology. Future research using experimental and/or longitudinal research approaches could provide evidence for the possible causal role of polyculturalism as indicated in these theoretical proposals.

Although the findings of the current study involve small effect sizes and should be seen as preliminary, the results indicate that one diversity ideology is negatively associated with dehumanization. The evidence does not yet point to that one diversity ideology’s role in reducing dehumanization, but there is now initial evidence to indicate how polycultural beliefs are conceptually opposed to specific subtle expressions of dehumanization. Indeed, “humanizing” IPs in the Philippines might mean, at least in small part, acknowledging their distinct cultural identities, norms, and practices. It also means emphasizing how they also share sociocultural experiences that connect them with non-IP Filipinos.

## Figures and Tables

**Table 1:** Summary of descriptive statistics for trait attributions for different groups.

	High Human Uniqueness/ High Human Nature Traits		High Human Uniqueness/ Low Human Nature Traits	
	M	SD	M	SD
Filipinos	4.61 ^a^	0.76	4.24 ^b^	0.90
Igorots	4.28 ^b^	0.85	4.44 ^a^	0.84
Lumads	4.27 ^b^	0.86	4.31 ^b^	0.89
Mangyans	4.21 ^c^	0.82	4.34 ^b^	0.82

Note: Means in one column that have different superscripts are statistically different from each other based on pairwise comparisons corrected using the Bonferroni method.

**Table 2:** Descriptive statistics of relative dehumanization indexes for the three IP groups.

	Igorots			Lumads			Mangyans		
	RDI-HHN	RDI-LHN	RDI-HHU	RDI-HHN	RDI-LHN	RDI-HHU	RDI-HHN	RDI-LHN	RDI-HHU
Mode	0.00	0.00	0.00	0.00	0.00	0.25	0.00	0.00	0.38
Mean	0.34	0.20	0.07	0.34	0.62	0.13	0.40	0.09	0.16
Standard Deviation	0.83	0.99	0.79	0.86	1.06	0.84	0.82	0.98	0.78
95% CI Mean Upper	0.41	0.28	0.14	0.42	0.15	0.21	0.47	0.18	0.22
95% CI Mean Lower	0.27	0.12	0.00	0.27	−0.03	−0.07	0.33	0.01	0.09
Minimum	−2.25	−3.40	−3.38	−3.25	−3.00	−3.88	−3.25	−2.75	−3.88
Maximum	3.50	4.50	3.50	3.25	4.50	3.13	2.75	4.50	2.50

**Table 3:** Summary of results of hierarchical regression analysis of predictors of RDI-HHNs of the three IP groups.

	Igorots				Lumads				Mangyans			
Predictors	Beta	95% CI (LL, UL)	Coll Stats		Beta	95% CI (LL, UL)	Coll Stats		Beta	95% CI (LL, UL)	Coll Stats	
			TOL	VIF			TOL	VIF			TOL	VIF
Age	0.07	−0.01, 0.06	1.00	1.00	0.08	−0.00, 0.07	0.99	1.01	0.06	−0.01, 0.06	1.00	1.00
Sex	0.03	−0.09, 0.20	0.95	1.05	0.03	−0.10, 0.20	0.95	1.06	0.04	−0.07, 0.22	0.95	1.06
OGK	−0.05	−0.09, 0.02	0.95	1.05	−0.12 **	−0.14, −0.02	0.96	1.05	−0.08	−0.11, 0.01	0.97	1.04
SDO	0.12 *	0.02, 0.18	0.85	1.18	0.10 *	0.01, 0.17	0.86	1.17	0.09	−0.00, 0.15	0.86	1.17
EGAL	0.13 **	0.04, 0.23	0.79	1.27	0.12 **	0.03, 0.24	0.79	1.27	0.12 *	−0.00, 0.22	0.79	1.27
ESSENRACE	0.07	−0.02, 0.14	0.87	1.15	0.08	−0.01, 0.16	0.87	1.15	0.05	−0.03, 0.12	0.87	1.15
Polyculturalism	−0.15 **	−0.31, −0.05	0.56	1.78	−0.19 ***	−0.37, −0.10	0.56	1.78	−0.20 ***	−0.36, −0.11	0.56	1.78
Multiculturalism	−0.05	−0.21, 0.08	0.55	1.80	−0.04	−0.20, 0.10	0.55	1.81	−0.01	−0.16, 0.12	0.55	1.82
*R*^2^ Model 1	0.04				0.03				0.03			
*R*^2^ Model 2	0.07				0.07				0.07			
*F* (8, 522)	4.58 ***				6.20 ***				4.59 ***			
Δ*R*^2^	0.03				0.04				0.04			
Δ*F* (2, 522)	8.62 **				11.98 ***				11.01 ***			

Note: OGK = outgroup knowledge, SDO = social dominance orientation, EGAL = egalitarianism, ESSENRACE = essentializing race, Beta = standardized beta, Coll stats = collinearity statistics, TOL = tolerance, VIF = variance inflation factor; * *p* < 0.05, ** *p* < 0.01, *** *p* < 0.001.

**Table 4:** Summary of results of hierarchical regression analysis of predictors of RDI-LHNs of the three IP groups.

	Igorots				Lumads				Mangyans			
Predictors	Beta	95% CI (LL, UL)	Coll Stats		Beta	95% CI (LL, UL)	Coll Stats		Beta	95% CI (LL, UL)	Coll Stats	
			TOL	VIF			TOL	VIF			TOL	VIF
Age	−0.04	−0.04, 0.01	1.00	1.00	−0.06	−0.04, 0.01	0.99	1.01	−0.04	−0.03, 0.01	1.00	1.00
Sex	0.01	−0.10, 0.12	0.95	1.05	0.06	−0.04, 0.17	0.95	1.06	0.01	−0.09, 0.11	0.95	1.06
OGK	0.07	−0.01, 0.07	0.95	1.05	0.09	−0.00, 0.08	0.96	1.05	0.07	−0.01, 0.07	0.97	1.04
SDO	−0.08	−0.11, 0.01	0.85	1.18	−0.03	−0.07, 0.04	0.86	1.17	−0.08	−0.10, 0.01	0.86	1.17
EGAL	−0.03	−0.10, 0.05	0.79	1.27	−0.09 *	−0.14, 0.00	0.79	1.27	−0.05	−0.11, 0.03	0.79	1.27
ESSENRACE	−0.01	−0.07, 0.05	0.87	1.15	−0.02	−0.07, 0.05	0.87	1.15	0.02	−0.04, 0.07	0.87	1.15
Polyculturalism	−0.13 *	−0.21, −0.01	0.56	1.78	−0.05	−0.13, 0.06	0.56	1.78	−0.02	−0.11, 0.07	0.56	1.78
Multiculturalism	0.02	−0.09, 0.12	0.55	1.80	0.00	−0.10, 0.11	0.55	1.81	−0.03	−0.13, 0.07	0.55	1.82
*R*^2^ Model 1	0.02				0.02				0.01			
*R*^2^ Model 2	0.03				0.03				0.02			
*F* (8, 522)	1.84				1.69				1.03			
Δ*R*^2^	0.01				0.00				0.00			
Δ*F* (2, 522)	3.26 *				0.44				0.55			

Note: OGK = outgroup knowledge, SDO = social dominance orientation, EGAL = egalitarianism, ESSENRACE = essentializing race, Beta = standardized beta, Coll stats = collinearity statistics, TOL = tolerance, VIF = variance inflation factor; * *p* < 0.05.

**Table 5:** Summary of results of hierarchical regression analysis of predictors of RDI-HHUs of the three IP groups.

	Igorots				Lumads				Mangyans			
Predictors	Beta	95% CI (LL, UL)	Coll Stats		Beta	95% CI (LL, UL)	Coll Stats		Beta	95% CI (LL, UL)	Coll Stats	
			TOL	VIF			TOL	VIF			TOL	VIF
Age	0.06	−0.01, 0.05	1.00	1.00	0.06	−0.01, 0.06	0.99	1.01	0.06	−0.01, 0.05	1.00	1.00
Sex	0.08	−0.00, 0.27	0.95	1.05	0.09 *	0.02, 0.30	0.95	1.06	0.09 *	0.01, 0.28	0.95	1.06
OGK	−0.03	−0.07, 0.03	0.95	1.05	−0.11 *	−0.13, −0.02	0.96	1.05	−0.06	−0.10, 0.01	0.97	1.04
SDO	0.08	−0.00, 0.14	0.85	1.18	0.08	−0.01, 0.15	0.86	1.17	0.05	−0.03, 0.12	0.86	1.17
EGAL	0.11 *	0.02, 0.20	0.79	1.27	0.08	−0.01, 0.19	0.79	1.27	0.09	−0.00, 0.18	0.79	1.27
ESSENRACE	0.11 *	0.02, 0.17	0.87	1.15	0.12 **	0.03, 0.19	0.87	1.15	0.10 *	0.01, 0.16	0.87	1.15
Polyculturalism	−0.21 ***	−0.36, −0.12	0.56	1.78	−0.22 ***	−0.38, −0.13	0.56	1.78	−0.22 ***	−0.37, −0.13	0.56	1.78
Multiculturalism	−0.07	−0.22, 0.05	0.55	1.80	−0.06	−0.22, 0.06	0.55	1.81	−0.05	−0.19, 0.07	0.55	1.82
*R*^2^ Model 1	0.04				0.05				0.03			
*R*^2^ Model 2	0.10				0.11				0.08			
*F* (8, 522)	7.36 ***				8.23 ***				6.78 ***			
Δ*R*^2^	0.06				0.06				0.06			
Δ*F* (2, 522)	17.44 ***				16.99 ***				16.76 ***			

Note: OGK = outgroup knowledge, SDO = social dominance orientation, EGAL = egalitarianism, ESSENRACE = essentializing race, Beta = standardized beta, Coll stats = collinearity statistics, TOL = tolerance, VIF = variance inflation factor; * *p* < 0.05, ** *p* < 0.01, *** *p* < 0.001.

## Data Availability

All data and materials used in the study are available from the author upon reasonable request.

## Supplementary Materials

Four supplementary tables that provide supporting statistical information can be downloaded at: https://www.mdpi.com/article/10.3390/bs16071142/s1, Table S1: Zero-order correlations for all variables in regression analyses; Table S2: Summary of complete hierarchical regression analyses of predictors of RDI-HHN of three IP groups; Table S3: Summary of complete hierarchical regression analyses of predictors of RDI-LHN of three IP groups; Table S4. Summary of complete hierarchical regression analyses of predictors of RDI-HHU of three IP groups.

## Abbreviations

The following abbreviations are used in this manuscript:

ANOVA	Analysis of Variance
HN	Human nature
HU	Human uniqueness
IP	Indigenous People
RDI-HHN	Relative dehumanization index involving high human nature traits
RDI-LHN	Relative dehumanization index involving low human nature traits
RDI-HHU	Relative dehumanization index involving high human uniqueness traits
