# Supplementary material for: Diversity Ideologies and the Subtle Dehumanization of Indigenous Peoples in the Philippines
Source: Behav Sci (Basel). 2026 Jul 7;16(7):1142. doi: 10.3390/bs16071142 (PMC13404204; doi:10.3390/bs16071142)
Supplement: Supplementary file 1 [file behavsci-16-01142-s001.zip › behavsci-4289125-supplementary.pdf]

Supplementary Table S1. *Zero-order Correlations for All Variables in Regression Analyses*

Variable	Pearson's *r*																	
	1	2	3	4	5	6	7	8	9	10	11	12	13	14	15	16	17	18
1. Age	—																	
2. Sex	.03	—																
3. OGK Igorot	.00	.13**	—															
4. OGK Lumad	-.08	.13**	.70***	—														
5. OGK Mangyan	-.01	.14**	.73***	.81***	—													
6. SDO	.02	-.10*	-.12**	-.07	-.02	—												
7. EGAL	-.01	.15***	.17***	.14***	.11*	-.21***	—											
8. ESSENRACE	.03	.05	.06	.08	.08	.28***	.11*	—										
9. POLY	-.01	-.05	.00	.03	-.03	-.10*	.32***	-.00	—									
10. MULT	-.03	.03	.04	.04	-.01	-.08	.35***	.11**	.64***	—								
11. RDI-HHN Igorot	.08	.05	-.04	-.05	-.02	.13**	.04	.11*	-.18***	-.11*	—							
12. RDI-HHN Lumad	.10*	.04	-.03	-.11*	-.05	.12**	.03	.11*	-.19***	-.12**	.77***	—						
13. RDI-HHN Mangyan	.07	.06	-.02	-.06	-.05	.10*	.04	.08	-.18***	-.10*	.85***	.82***	—					
14. RDI-LHN Igorot	-.05	-.13**	-.01	.01	-.04	-.08	.01	-.13**	.25***	.17***	-.49***	-.43***	-.44***	—				
15. RDI-LHN Lumad	-.05	-.13**	.02	.06	.04	-.10*	.04	-.13**	.23***	.17***	-.36***	-.54***	-.41***	.77***	—			
16. RDI-LHN Mangyan	-.05	-.13**	-.02	.04	.00	-.06	.01	-.13**	.22***	.16***	-.44***	-.47***	-.50***	.88***	.84***	—		
17. RDI-HHU Igorot	.07	.10	-.01	-.03	.01	.12**	.02	.14**	-.24***	-.17***	.84***	.68***	.72***	-.89***	-.68***	-.78***	—	
18. RDI-HHU Lumad	.08	.10	-.03	-.09*	-.05	.12**	-.01	.14**	-.24***	-.17***	.63***	.85***	.68***	-.70***	-.90***	-.77***	-.77***	—
19. RDI-HHU Mangyan	.07	.11	.00	-.06	-.03	.09*	.01	.13**	-.23***	-.16***	.72***	.73***	.84***	-.78***	-.74***	-.89***	-.87***	-.84***

Note: OGK = outgroup knowledge, SDO = social dominance orientation, EGAL = egalitarianism, ESSENRACE = essentializing race, POLY = polyculturalism, MULT = multiculturalism, RDI-HHN = relative dehumanization index-high human nature traits, RDI-LHN = relative dehumanization index-low human nature traits, RDI-HHU = relative dehumanization index-high human uniqueness traits,; $^{*}p < .05$, $^{**}p < .01$, $^{***}p < .001$

Supplementary Table S2. *Summary of Complete Hierarchical Regression Analyses of Predictors of RDI-HHN of Three IP Groups*

Predictors	Igorots		Lumads		Mangyans	
	Beta	95% CI (LL, UL)	Beta	95% CI (LL, UL)	Beta	95% CI (LL, UL)
**Model 1**						
Age	.07	-.01, .06	.08	-.00, .07	.06	-.01, .06
Sex	.05	-.07, .23	.05	-.07, .24	.06	-.04, .25
Outgroup knowledge	-.04	-.08, .03	-.11**	-.14, -.02	-.06	-.10, .02
SDO	.13**	.03, .19	.11*	.01, .18	.10*	.01, .16
Egalitarianism	.06	-.03, .16	.05	.05, .15	.05	-.05, .14
Essentializing race	.06	-.02, .14	.08	-.01, .16	.05	-.04, .13
R^2 Model 1	.04		.03		.03	
F (6, 523)	3.15**		4.10***		2.36*	
**Model 2**						
Age	.07	-.01, .06	.08	-.00, .07	.06	-.01, .06
Sex	.03	-.09, .20	.03	-.10, .20	.04	-.07, .22
Outgroup knowledge	-.05	-.09, .02	-.12**	-.14, -.02	-.08	-.11, .01
SDO	.12*	.02, .18	.10*	.01, .17	.09	-.00, .15
Egalitarianism	.13**	.04, .23	.12**	.03, .24	.12*	-.00, .22
Essentializing race	.07	-.02, .14	.08	-.01, .16	.05	-.03, .12
Polyculturalism	-.15**	-.31, -.05	-.19***	-.37, -.10	-.20***	-.36, -.11
Multiculturalism	-.05	-.21, .08	-.04	-.20, .10	-.01	-.16, .12
R^2 Model 2	.07		.07		.07	
F (8, 522)	4.58***		6.20***		4.59***	
ΔR^2	.03		.04		.04	
ΔF (2, 522)	8.62**		11.98***		11.01***	

Note: SDO = social dominance orientation; *p < .05, **p < .01, ***p < .001.

Supplementary Table S3. *Summary of Complete Hierarchical Regression Analyses of Predictors of RDI-LHN of Three IP Groups*

	Igorots		Lumads		Mangyans	
Predictors	Beta	95% CI (LL, UL)	Beta	95% CI (LL, UL)	Beta	95% CI (LL, UL)
**Model 1**						
Age	-.04	-.04, .01	-.06	-.04, .01	-.03	-.03, .01
Sex	.02	-.08, .14	.06	-.03, .18	.01	-.09, .11
Outgroup knowledge	.07	-.01, .07	.09	-.00, .08	.07	-.01, .07
SDO	-.07	-.11, .01	-.02	-.07, .04	-.08	-.10, .01
Egalitarianism	-.07	-.12, .02	-.10*	-.15, -.01	-.07	-.11, .02
Essentializing race	-.01	-.07, .06	-.02	-.07, .74	.02	-.04, .07
R^2 Model 1	.02		.02		.01	
F (6, 523)	1.35		2.12		0.55	
**Model 2**						
Age	-.04	-.04, .01	-.06	-.04, .01	-.04	-.03, .01
Sex	.01	-.10, .12	.06	-.04, .17	.01	-.09, .11
Outgroup knowledge	.07	-.01, .07	.09	-.00, .08	.07	-.01, .07
SDO	-.08	-.11, .01	-.03	-.07, .04	-.08	-.10, .01
Egalitarianism	-.03	-.10, .05	-.09*	-.14, .00	-.05	-.11, .03
Essentializing race	-.01	-.07, .05	-.02	-.07, .05	.02	-.04, .07
Polyculturalism	-.13*	-.21, -.01	-.05	-.13, .06	-.02	-.11, .07
Multiculturalism	.02	-.09, .12	.00	-.10, .11	-.03	-.13, .07
R^2 Model 2	.03		.03		.02	
F (8, 521)	1.84		1.69		1.03	
ΔR^2	.01		.00		.00	
ΔF (2, 521)	3.26*		0.44		0.55	

Note: SDO = social dominance orientation; *p < .05, **p < .01, ***p < .001.

Supplementary Table S4. *Summary of Complete Hierarchical Regression Analyses of Predictors of RDI-HHU of Three IP Groups*

	Igorots		Lumads		Mangyans	
Predictors	Beta	95% CI (LL, UL)	Beta	95% CI (LL, UL)	Beta	95% CI (LL, UL)
**Model 1**						
Age	.06	-.01, .06	.06	-.01, .06	.06	-.01, .06
Sex	.11*	.03, .31	.12**	.06, .35	.09*	.05, .32
Outgroup knowledge	-.02	-.06, .04	-.11*	-.13, -.01	-.05	-.09, .03
SDO	.10*	.00, .15	.09*	.00, .16	.07	-.02, .13
Egalitarianism	.01	-.08, .10	-.01	-.11, .08	-.00	-.09, .09
Essentializing race	.11*	.02, .17	.12*	.03, .19	.10*	.01, .16
R^2 Model 1	.04		.05		.03	
F (6, 523)	3.77***		5.03***		3.29**	
**Model 2**						
Age	.06	-.01, .05	.06	-.01, .06	.06	-.01, .05
Sex	.08	-.00, .27	.09*	.02, .30	.09*	.01, .28
Outgroup knowledge	-.03	-.07, .03	-.11*	-.13, -.02	-.06	-.10, .01
SDO	.08	-.00, .14	.08	-.01, .15	.05	-.03, .12
Egalitarianism	.11*	.02, .20	.08	-.01, .19	.09	-.00, .18
Essentializing race	.11*	.02, .17	.12**	.03, .19	.10*	.01, .16
Polyculturalism	-.21***	-.36, -.12	-.22***	-.38, -.13	-.22***	-.37, -.13
Multiculturalism	-.07	-.22, .05	-.06	-.22, .06	-.05	-.19, .07
R^2 Model 2	.10		.11		.08	
F (8, 521)	7.36***		8.23***		6.78***	
ΔR^2	.06		.06		.06	
ΔF (2, 521)	17.44***		16.99***		16.76***	

Note: SDO = social dominance orientation; *$p < .05$, **$p < .01$, ***$p < .001$.
